# Comparative analysis of state-level policy responses in global health governance: A scoping review using COVID-19 as a case

**DOI:** 10.1371/journal.pone.0313430

**Published:** 2024-11-07

**Authors:** Fengyuan Tang, Wenqianzi Yang, Weijia Wu, Yewen Yao, Yi Yang, Qiyi Zheng, Baheti Maireyi, Shengxuan Jin, Hengjin Dong

**Affiliations:** 1 Department of Science and Education of the Fourth Affiliated Hospital and Center for Health Policy Studies, School of Public Health, Zhejiang University School of Medicine, Hangzhou, Zhejiang, China; 2 Health Management Research Center, School of Public Health, Southeast University, Nanjing, Jiangsu, China; University of Zagreb School of Medicine: Sveuciliste u Zagrebu Medicinski fakultet, CROATIA

## Abstract

**Background:**

States are key actors in global health governance, particularly in the prevention and control of infectious diseases. The emergence and re-emergence of infectious diseases in recent decades pose profound challenges to global health security. As the first coronavirus pandemic, the COVID-19 caused significant damage worldwide, but responses and outcomes varied greatly among states. Using COVID-19 as an example, this study aims to compare the policies and measures implemented by different states during the COVID-19 pandemic and to synthesize experiences to strengthen global health governance for future infectious disease crises.

**Methods:**

We used Arksey and O’Malley’s five-stage scoping review framework and PRISMA methodology was used for literature search and decision on relevant studies. English databases were searched using combinations of keywords and articles examining COVID-19 prevention and control policies in representative countries were included. A comparative analysis across these four states (United States, Sweden, India, and Nigeria) was then conducted to analyse the differences, rationale, and challenges of the approaches taken by these states.

**Results:**

A total of 36 studies were included in the analysis. The management of the COVID-19 by states is divided into two main categories: domestic governance and international governance. Domestically, the United States and India have taken more measures, yet notable disparities in infection source control, transmission interruption, vulnerable population protection, collaborative governance, and so on were observed among all four states. Globally, the United States and Sweden were more proactive in international governance, and all four states have variations in their adherence to global regulations, information sharing, resource distribution, and cooperative engagement.

**Conclusions:**

Significant disparities occurred during the response to early COVID-19 in four states, which may be due to differences in politics, economy, and culture. To prevent and mitigate the impact of infectious diseases, states should, in future, prioritize solidarity and cooperation, and improve governance domestically and internationally based on national contexts and global health principles.

## Background

Global health governance (GHG) can be defined as the process of promoting cooperation in the field of global health, formulating and implementing binding international regulations to better address global health crises, promote health equity, and ultimately achieve global health [1]. GHG involves a number of actors to address factors crossing even ignoring geographical boundaries from a multi-sectoral perspective [2]. The actors of GHG include states, international government organizations, non-governmental organizations, civil society, private sectors, among which states are the most important actors for the reason that others depend on or originate from certain states [1].

Through coordinating international resources and responses, facilitating the equitable distribution of medical supplies and knowledge, and ensuring that all states can effectively manage health emergencies, one of the key goals of GHG is to contain the spread of infectious diseases, which can easily traverse geographical boundaries with the rapid advancement of globalization and potentially trigger global pandemics. In recent decades, the outbreaks of infectious diseases such as Severe Acute Respiratory Syndrome (SARS), Middle East Respiratory Syndrome (MERS), and Ebola virus disease have severely undermined global health security. In early 2020, Coronavirus Disease 2019 (COVID-19) broke out, marking the first coronavirus infection to be called a "pandemic" due to its alarming spread and severity. Despite the adoption of numerous policies and measures by states to combat COVID-19, the results have been less than ideal, with the number of cases and deaths increasing rapidly. As of January 2024, there have been 774 million confirmed cases of COVID-19 and 7 million deaths [3]. The COVID-19 pandemic exposed weaknesses and inequities in governance and severely impacted medical and health systems worldwide, resulting in significant losses to the global community.

As the primary actor in GHG, states adopted their respective policies and measures during the COVID-19 pandemic. These different responses resulted in a spectrum of outcomes in terms of disease burden, mortality rates, and the long-term socio-economic effects on the populations. Although researchers have attempted to analyze policies and their effects in different states, many states with different economic development status and effect of COVID-19 prevention and control are neglected [4,5], and the cross-border characteristic of infectious diseases has been ignored [6]. Therefore, it is crucial to select examples and analyze the differences and challenges of their governments’ policies in responding to COVID-19 from a global health perspective and put forward relevant suggestions to improve the global health governance of infectious disease pandemics.

## Methods

Economic development can influence resource allocation for healthcare systems and emergency responses [7], while the effect of COVID-19 control is a key objective indicator for evaluating control policies and measures performed. Thus, to reveal how resource constraints and public health strategies interact, we divided all states into groups based on economic development and the effect of COVID-19 control. According to the 2023 World Bank income classification based on Gross National Income (GNI) per capita, states were classified into high-income (GNI per capita above USD 13,845) or low-and middle-income groups (GNI per capita USD 13,845 or lower) [8]. The effect of COVID-19 control was illustrated by cumulative number of confirmed cases and deaths [9]. The data of individual states and income group, namely low-and middle-income or high-income states, from the website Our World in Data [10] was used to categorize states according to their population-adjusted cumulative number of cases and deaths in the early stage. States with cumulative cases or deaths exceeding the threshold value for their respective income group (e.g., 436.9 cumulative cases and 25.7 cumulative deaths per million in high-income states, 13.4 cumulative cases and 0.9 cumulative deaths per million in low-and middle-income states, as of April 1, 2020) were categorized as having "more cases and deaths," while those with values equal to or below these thresholds were categorized as having "fewer cases and deaths." Accordingly, four distinct groups were identified: 1) High-income states with more cases and deaths; 2) High-income states with fewer cases and deaths; 3) Low-and middle-income states with more cases and deaths; 4) Low-and middle-income states with fewer cases and deaths.

To facilitate the presentation and comparison of prevention and control policies, we selected one example from each of the four groups: 1) the United States, a high-income country with high absolute numbers of COVID-19 cases and deaths, illustrating the challenges of pandemic response within decentralized governance systems; 2) Sweden, a high-income state with fewer cases and deaths, known for its less restrictive approach and reliance on voluntary measures, providing a unique case study on balancing public health with individual freedoms; 3) India, a low-and middle-income country with a high burden of cases and deaths, highlighting the challenges of pandemic management in resource-constrained settings and the importance of international collaboration; and 4) Nigeria, a low- and middle-income country with relatively lower reported cases and deaths, offering insights into global health governance through regional collaboration and local innovations, particularly within the African context.

As a crucial period in preventing and controlling infectious diseases, the early stage is the focus of our research, that is, the interval from the first reported case to point where new cases began to consistently decline. If the outbreak was not effectively controlled and the number of daily new cases did not decrease significantly, the endpoint of our analysis was set at the time when policies are eased, such as lifting lockdown measures [10–13]. For the policies related to other states or vaccine promotion, the scope of the research was extended to the full duration of the pandemic.

A comprehensive search of three databases (Medline, Embase and Web of Science) was conducted on 19 November 2023. Literature surveillance alerts were received on a weekly basis until 31 December 2023. Inclusion of relevant literature was contingent upon the fulfilment of the following criteria: 1) Language was English and publication dates were from January 2020 to December 2023; 2) Studies were conducted in one or more relevant representative countries; 3) Studies were about early policies and measures to prevent and control COVID-19. Literatures were excluded they were letter to the editor, opinion, editorial, conference proceeding, interview, or if the research did not address the selected representative country or the early governance of COVID-19. In addition, official public health websites were consulted as supplementary sources for policy information. In particular, the websites of the Centers for Disease Control and Prevention (U.S.), the Government Office of Sweden and Public Health Agency of Sweden, the Nigeria Center for Disease Control and Prevention, and the Ministry of Health and Family Welfare (MoHFW, India) were reviewed.

We conducted a qualitative scoping review based on Arksey and O’Malley’s five-stage scoping review framework to compare COVID-19 governance policies and measures across these four states by compiling and synthesizing information. This allowed us to evaluate the strengths and weaknesses of these approaches and explore the possible reasoning underlying them. Ultimately, we offer suggestions for enhancing the management of infectious diseases within the framework of global health governance. The methodological details of this study including scoping review framework, literature search strategy, screening process, and data management approach are presented in the Supplementary Material.

## Results

**Fig 1:**
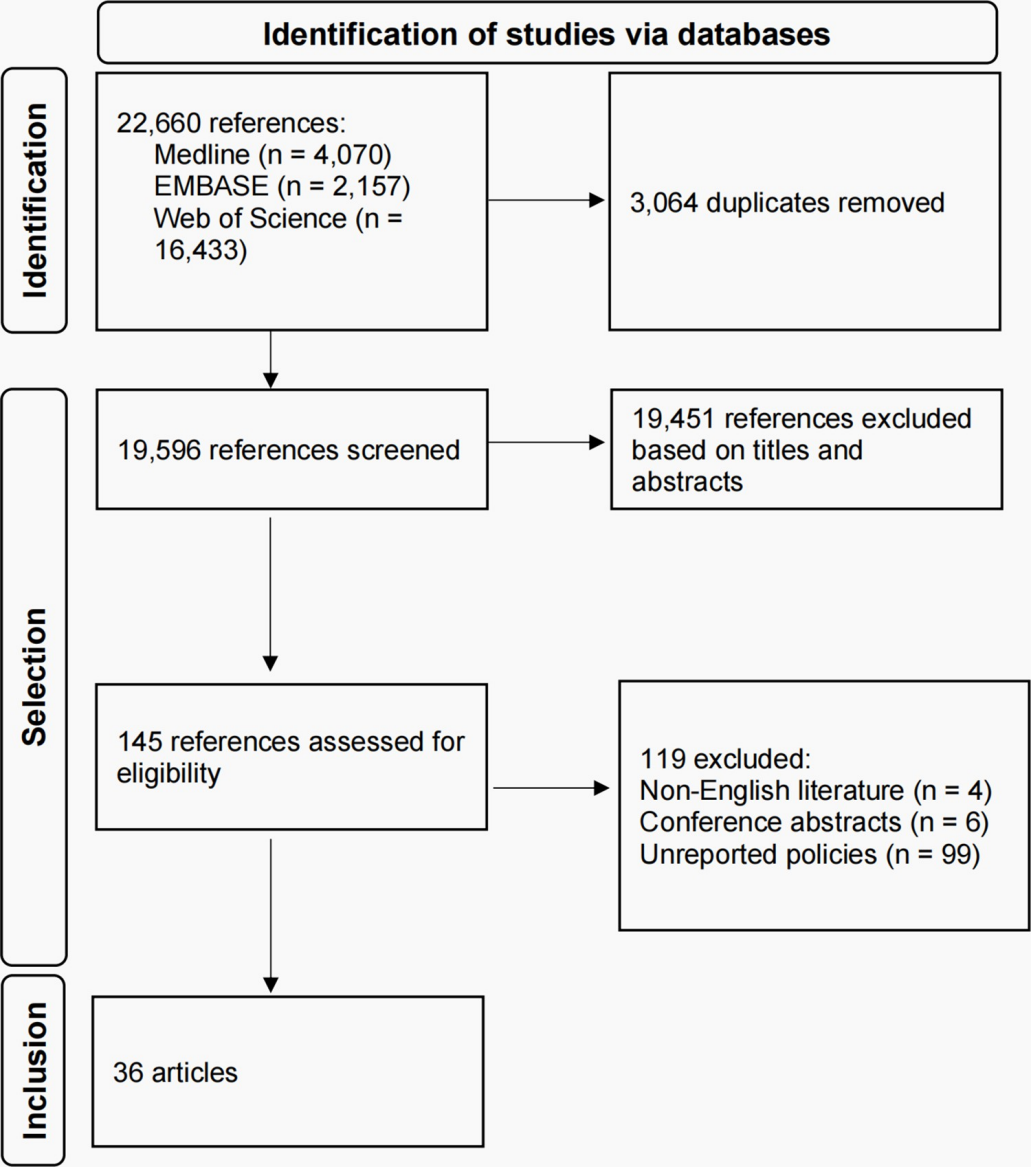
PRISMA flowchart of state-level policy responses in the early stage of COVID-19 pandemic.

**Fig 2:**
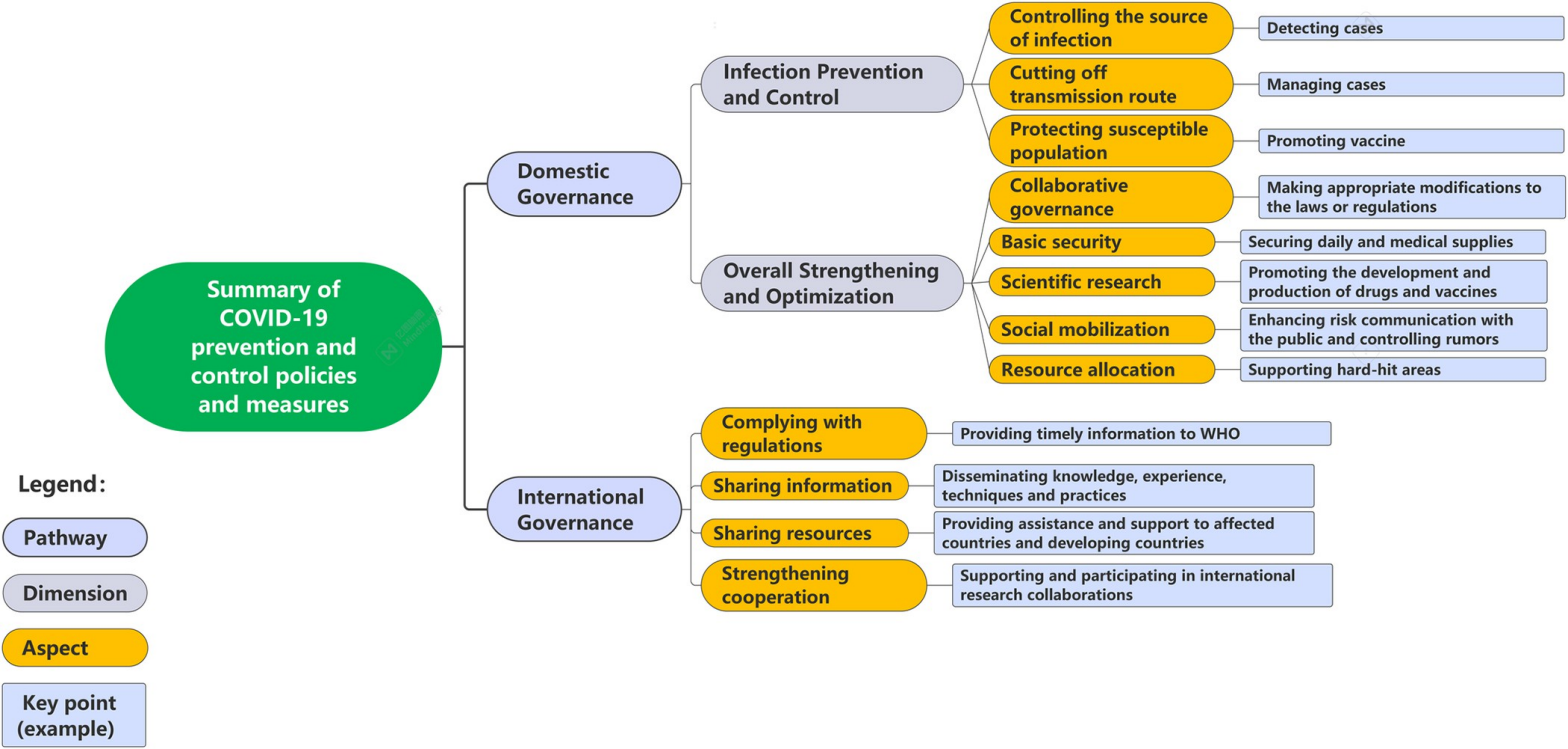
The summary of COVID-19 governance policies and measures.

A total of 22,660 references were identified, of which 3,064 duplicates were removed through the use of the NoteExpress software. Among the 19,596 titles and abstracts that were screened, 19,451 were excluded because they did not meet the inclusion criteria. A total of 145 full-text papers were then assessed for their eligibility, of which 119 were excluded according to exclusion criteria and 36 studies were included at last. **Fig 1** provides a comprehensive illustration of the systematic process of identifying, screening, and including relevant studies through the PRISMA flowchart. The majority of the included studies employed descriptive and qualitative research methodologies by listing relevant policies conducted. By summarizing COVID-19 governance practices in different states, we found that there are two pathways for states to conduct the governance of COVID-19: 1) domestic governance limited to the state itself and 2) international governance related to other actors abroad. Domestic governance refers to a state’s efforts to prevent and control infectious disease outbreaks, protect public health, and ensure social stability and development within its borders. International governance involves global cooperation among states and other actors to combat infectious diseases and promote human health, following the principles and requirements of the global health governance framework. The main aspects of domestic and international governance and key points are shown in **Fig 2**.

As SARS-CoV-2 is a novel pathogen, non-pharmaceutical interventions are the primary means of controlling the spread of the disease at the onset of an outbreak [14]. Therefore, the domestic governance measures include: 1) “Infection Prevention and Control”, which includes controlling the source of infection, cutting off the transmission route, and protecting the susceptible population; and 2) “Overall Strengthening and Optimization” including collaborative governance, basic security, scientific research, social mobilization, and resource allocation. While achieving domestic governance, states also carried out international governance containing complying regulations, sharing information and resources, and strengthening cooperation [15–22].

### Domestic governance

In terms of domestic governance, differences are centred on: 1) Controlling infection source; 2) cutting off transmission route; 3) protecting susceptible population; 4) collaborative governance and 5) other aspects. As states with high numbers of patients and deaths from COVID-19, the United States and India have taken relatively more preventive and control measures in the domestic governance of COVID-19. In accordance with the above-mentioned dimensions, aspects and key points, the implementation of domestic governance in the representative states is presented in **Table 1** and further information can be found (**S1 Table in S1 File**).

**Table 1:** Domestic governance policies and measures in the early COVID-19 pandemic among four states.

Dimension	Aspect	Key point	Detail	Representative state			
				High-income state with more cases and deaths	High-income state with fewer cases and deaths	Low-and middle-income state with more cases and deaths	Low-and middle-income state with fewer cases and deaths
				the U.S.	Sweden	India	Nigeria
Infection Prevention and Control	Controlling the source of infection	Detecting cases	Risk screening in key institutions	√	√	NA	√
			Risk screening in key populations	√	√	√	√
		Tracing contacts		√	√	√	√
		Improving detection capability		√	√	√	√
		Locking down risk areas		√	NA	√	√
	Cutting off transmission route	Managing cases		√	√	√	√
		Managing potential cases		√	√	√	√
		Supervising personal protection		√	√	√	√
		Restricting the movement of people		√	√	√	√
		Social distancing	Controlling or cancelling mass gatherings	√	√	√	√
			Closing recreation places, workplaces, schools	√	√	√	√
			Issuing a stay-at-home order	√	√	√	√
	Protecting susceptible population	Protecting key populations	Protecting the safety of older people, pregnant women, children and other vulnerable groups	√	√	√	√
			Protecting the safety of health workers	√	√	√	√
		Promoting vaccine		√	√	√	√
		Sustaining medical services	Setting up COVID-19 designated hospitals	√	NA	√	NA
			Carrying out telemedicine	√	√	√	NA
Overall Strengthening and Optimization	Collaborative governance	Making appropriate modifications to the laws or regulations		√	√	√	NA
	Basic security	Securing daily and medical supplies		√	√	√	√
		Providing palliative support		√	√	√	√
		Protecting mental health		√	NA	√	NA
		Improving medical care		√	√	√	√
	Scientific research	Promoting the development and production of drugs and vaccines		√	NA	√	√
	Social mobilization	Enhancing risk communication with the public and controlling rumours		√	√	√	√
		Carrying out public health education		√	√	√	√
		Mobilizing the private sector and communities		√	√	√	√
	Resource allocation	Expanding the number and capacity of medical institutions		√	√	√	√
		Supporting hard-hit areas		√	√	NA	√

√: Corresponding measures were taken; NA: Not available.

The table presents an overview of domestic governance policies and measures implemented in the early stage of COVID-19 pandemic in the four represented states.

#### Controlling the source of infection

States vary in their methods of case detection, contact tracing, and lockdown. Screening risk in key institutions was adopted by all states except India. During community transmission, Sweden stopped contact tracing while other states continued to do so [11], meanwhile, India and the U.S. released digital tracing apps to assist with tracing [23]. Significant differences existed in the lockdown policies of the four states. Nigeria enforced a strict lockdown in the most affected areas, India implemented a nationwide lockdown, the U.S. implemented only a 15-day voluntary national lockdown, and Sweden did not impose a lockdown [13,23].

#### Cutting off transmission route

Regarding case management, Nigeria isolated confirmed cases but the U.S. and Sweden rarely isolated cases but advised people to self-isolate at home. It’s difficult to determine whether India has quarantined patients. Nigeria and India both implemented mandatory mask-wearing protocols in public areas [10,24], while the U.S. had no similar requirements and Sweden only advised mask usage for health care and elderly care settings [25]. Domestic and international travel restrictions were implemented by Nigeria and India, while the U.S. and Sweden focused on reducing international travel [26]. Regarding social distancing, all states aimed to control or cancel mass gatherings, except Sweden, where schools for children up to 16 years and public spaces remained open [26].

#### Protecting susceptible population

There have been several ways to protect vulnerable populations. The U.S. focused on health equity issues among disproportionately affected populations, such as racial and ethnic minorities [27]. Sweden prioritized the protection of the elderly and hospitalized individuals by implementing measures such as banning visits to nursing homes and hospitals [28]. The Ministry of Health and Family Welfare in India has published health advice and guidelines for vulnerable groups. Additionally, they have introduced an insurance scheme for health workers fighting COVID-19. In Nigeria, the focus was on the poor and vulnerable, providing food and cash assistance. In order to maintain medical services, the United States and India constructed new hospitals and implemented telemedicine. On the other hand, Sweden and Nigeria did not increase their number of hospitals, and Nigeria failed to establish efficient telemedicine methods.

#### Collaborative governance

Governments implemented different policies from the perspective of the whole society to combat infectious diseases. Regarding the leadership form, the U.S. established a public health emergency management system with a vertical hierarchy of “federal–state–local”, and a horizontal division of labour between governments, private sectors, and volunteer groups [5]. The Public Health Agency of Sweden served as the national coordinating body, which had a broad responsibility for public health and regions were autonomous [6]. For India, the state headquarters coordinates technology and resources for local governments, while the local administration operates independently [26]. Nigeria inaugurated the Presidential Task Force (PTF) on COVID-19 to coordinate the state’s multisectoral intergovernmental response [29].

#### Other aspects

In terms of basic security, the Food and Drug Administration (FDA) has issued guidance to expand the availability of digital health therapeutic devices for psychiatric disorders in the United States [30], but Nigeria and Sweden have implemented relatively few measures to protect the mental health of their populations. Compared with other states, the United States and India have done more work on COVID-19-related scientific research [5]. We could not find specific information on India’s support for the hard-hit areas. However, other states have made efforts to provide aid to these regions.

### International governance

In terms of international governance, differences are centred on: 1) regulation compliance; 2) information sharing; 3) resource distribution; 4) cooperation strengthening. India and Nigeria have taken fewer steps towards international governance of COVID-19 compared to high-income states like the United States and Sweden. In accordance with the above-mentioned aspects and key points, the implementation of international governance in the representative states is presented in **Table 2** and further information can be found (**S2 Table in S1 File**).

**Table 2:** International governance policies and measures in the early COVID-19 pandemic among four states.

Aspect	Key point	Representative state			
		High-income state with more cases and deaths	High-income state with fewer cases and deaths	Low-and middle-income state with more cases and deaths	Low-and middle-income state with fewer cases and deaths
		the U.S.	Sweden	India	Nigeria
Complying with regulations	Providing timely information to WHO	√	√	NA	NA
	Reasonable control of international traffic and trade	√	√	√	√
	Improving the prevention and control capacity of entry points	√	NA	√	√
Sharing information	Disseminating knowledge, experience, techniques and practices	√	√	NA	NA
	Referring to other countries’ experience in prevention and control	√	NA	NA	√
Sharing resources	Providing assistance and support to affected countries and developing countries	√	√	√	NA
	Supporting organizations that promote health governance	√	√	NA	NA
	Reducing trade barriers and maintaining trade in essential goods	NA	√	NA	NA
	Distributing resources such as medicines and vaccines equitably	√	√	√	NA
Strengthening cooperation	Supporting and participating in international research collaborations	√	NA	√	NA
	Strengthening cooperations with international governmental organizations, Non-Governmental Organizations and the private sectors	√	√	√	√
	Promoting partnerships, long-term cooperation mechanisms and cooperation with regional health organizations	√	√	√	√

√: Corresponding measures were taken; NA: Not available.

The table presents an overview of international governance policies and measures implemented in the early stage COVID-19 pandemic in the four represented states

#### Complying with regulations

When it comes to meeting regulatory requirements, it is crucial to promptly supply information to the World Health Organization (WHO). The U.S. strengthened IHR reporting and recommended people to avoid nonessential travel. Simultaneously, the US implemented border closures with Mexico and Canada and tightened travel restrictions for individuals from other nations with high rates of transmission [13]. Sweden was in continuous communication with the WHO leadership and didn’t recommend non-essential travel to other states. The information we gathered was insufficient to demonstrate that India and Nigeria were able to report information to the WHO promptly.

#### Sharing information

Regarding information sharing, Nigeria and the U.S. participated in the China-WHO Joint expert team and conducted field visits in China, the U.S. also analysed the experiences of other states to determine the effectiveness of the surveillance and containment measures [31]. The United States made important contributions in sharing viral genes. Sweden participated in work conducted by the EU Health Security Committee to enhance the sharing of information and coordination. India’s efforts to share knowledge and technology may be limited.

#### Distributing resources

Distributing resources is another important aspect of international governance. The United States provided support to Centres for Disease Control and Prevention (CDC) offices in affected states and set aside funds for international aid. Sweden donated COVID-19 vaccines to low- and middle-income states and contributed both political and financial support to the WHO and other multilateral organizations [32]. At the same time, Sweden made efforts to improve the functioning of the European single market and lift newly imposed export restrictions [33]. Except for Sweden, other states have taken less action to reduce trade barriers and maintain trade. India donated masks and other medical supplies to China and pledged nearly 100 million hydroxychloroquine tablets to Russia [10]. Nigeria has done relatively little to share resources.

#### Strengthening cooperation

The U.S. participated in therapeutic and vaccine clinical trials and broadened global respiratory surveillance activities with the WHO [34]. Sweden supported the UN appeal and the "Team Europe" approach to support partner states and worked with the EU and other actors to combat rumours and disinformation. India has partnered with AstraZeneca to produce large quantities of COVID-19 vaccine for the COVID-19 Vaccines Global Access (COVAX) program. Additionally, a fund named the COVID Fund for South Asian Association for Regional Cooperation (SAARC) States has been established [35]. Nigeria led infection prevention and control training in Africa in collaboration with the Africa COVAX [36].

## Discussion

### Policy orientation

Different states may have varying policy objectives and approaches for preventing and controlling COVID-19. These can be broadly classified into containment and mitigation strategies, as well as intermediate strategies in between [10]. The United States has implemented relatively lenient mitigation policies. This may be due to the federal system in place, which has limited the state’s ability to take a unified response. Moreover, the unstable stock market has called for a composed and calculated approach, and Americans are generally more distrustful of the government, particularly the federal government, resulting in obstacles to implementing containment policies [37]. Sweden also implemented relatively lax policies because it aimed to implement sustainable policies that could be executed over the long term, while still maintaining strict protection of basic rights [6,38]. This may indirectly explain Sweden’s “negative” stance on risk monitoring and contact tracing. Both India and Nigeria once implemented strict containment strategies in the early stage of the pandemic, however, the two states abandoned containment policies later which is primarily due to its high cost and lack of effectiveness. For instance, India’s stringent containment policies failed to “flatten the curve”. As a result, hospitals and healthcare workers were stretched to their limits, and the normal lives of people and the national economy were severely affected [39].

### Domestic governance

#### Controlling infection source

In terms of controlling the source of infection, ’detection’ and ’lockdown’ are two important aspects. The United States faced many challenges with testing, including delays in test reporting, shortages of testing kits and supplies, and disproportionate distribution of testing sites [5,40]. The limited availability of tests nationwide may be due to the CDC’s unsuccessful attempt to develop its own test and stringent government regulations on third-party test approval [41,42]. Similarly, India lacked sufficient risk monitoring at crucial sites, and Nigeria also faced issues with inadequate testing [43,44]. India and the United States used Internet technology to develop apps that can track close contacts to reduce the burden of manpower. However, these apps raised legitimate public concerns about privacy, data protection, and civil liberties [23]. Sweden did not implement a formal compulsory lockdown or state of emergency during peacetime due to constitutional limitations. However, despite the lack of a mandatory lockdown, many Swedes followed the government’s recommendations and changed their behaviours to prevent the spread of COVID-19, resulting in what was referred to as a ’virtual lockdown’ [6,25]. Although the lockdown was only implemented in the three most affected areas, Nigeria did not effectively implement it due to the fact that many Nigerians are self-employed and were unable to cope financially [43]. Nigeria reopened before the outbreak was fully under control, and so did the United States and India [45]. In the United States, the effective implementation of containment policies was hindered by the lack of manpower in both federal and local governments. Additionally, American communities were sparsely populated and rarely walled, which further complicated the situation [46]. When it comes to India, it may be counterintuitive that the containment strategy with nationwide lockdown has not achieved the desired results, which may be closely related to factors such as high levels of poverty and mobility, low levels of education of the population, and inadequate healthcare systems. These are common challenges faced by developing states like India and Nigeria [39].

#### Cutting off transmission route

The next step is cutting off the transmission routes of the virus. For Nigeria, the supervision of self-isolation of people was too lax [47] and there were cases of neglect and even rejection of patients in health facilities [43], which may be related to the backward economic foundation and lack of health resources in developing states [48]. Although Nigeria implemented strict policies, compliance with preventive guidelines was reportedly zero in some states due to the nonchalant attitude of both ordinary people and public figures [43]. Unlike Nigeria, Sweden, being a developed state, has sufficient medical resources to control the spread of infections, even during a pandemic like COVID-19 [28]. In regards to personal protection monitoring, India and Nigeria have stricter regulations, followed by Sweden. The United States, on the other hand, has more lenient regulations. The lack of personal protection supervision, such as mask mandates in the United States and Sweden increased the risk of infection in the population and hindered efforts to cut off transmission routes.

#### Protecting susceptible population

Almost all states have neglected vulnerable groups, including the United States and Sweden, which we focus on as examples because the challenge is more severe in developing states. Though equity is a fundamental principle of Swedish social systems [28], mitigation policies required mild patients to isolate at home [11], which made people living in poor conditions difficult to separate from other family members [6,11,28]. Similarly, in the United States, ethnic minorities face a higher risk of contracting the virus. Additionally, some states did not adequately protect their health workers, allowing them to continue working even after testing positive [5,23]. The inequalities may be due to limited access to health services, difficulty adhering to physical distancing recommendations, underlying health disparities, and intersecting stigmas [49]. In India, prenatal care and childhood immunizations were reduced, and the virus spread rapidly among the poor [10,39]. Moreover, the vaccination rate in India has been sluggish, with just about 2% of the population having received both doses of the vaccine even after four months since the launch of the vaccination campaign. Many states like India and Nigeria implemented strict transportation limitations or even a complete shutdown of transportation, affecting the satisfaction of necessary medical needs, especially for chronic patients [50] and leading to avoidable spread of COVID-19 [10], which highlighted the advanced nature of Sweden’s sustainable policy.

#### Collaborative governance

At the macro level, the U.S. government pursued a competitive, private-sector approach, leaving states and localities to fend for themselves. Unfortunately, there has been no coordinated implementation of policies [23], which has caused the price of medical resources to rise [45]. Federal and local conflicts were largely due to the fact that the U.S. Constitution places local responsibility for public health, while the federal government is primarily responsible for the spread of disease between states and internationally [37], and so is Sweden [38]. Therefore, achieving a high degree of coordination and unity in epidemic prevention and control is challenging for Sweden. However, Swedish experts, rather than politicians, have a voice, which has made Sweden’s prevention and control policy more scientific [11]. The issue of state incoordination was also present in Nigeria, which worsened the movement of people between states [43]. In India, the Ministry of Health and Family Welfare was responsible for overseeing national awareness and guideline implementation, while individual states managed local information dissemination independently. Districts coordinated with departments to streamline efforts in pandemic control, resulting in a structured approach that optimizes coordination [26,39]. Overall, India’s decision-making during the pandemic has been focused on the national level. India’s leadership model has demonstrated effective decentralized control in the pandemic response [13], but national-level management needs further harmonization.

#### Basic security

In terms of basic security, while the United States utilized the Defense Production Act to guarantee the production and distribution of essential goods, the implementation was limited in scope and duration, which failed to meet the demands [23]. Simultaneously, Nigeria faced a severe shortage of medical resources and other essential materials, resulting in panic hoarding [43], which was caused by inadequate funding and bureaucracy [44]. All states provided palliative support to individuals and enterprises. However, for the United States, additional stimulus funding was not passed between April and November 2020 [23]. Although Nigeria implemented palliative policies, the eligibility criteria were so strict that the scheme was largely ineffective [47,51]. Medical personnel in India have faced reduced salaries and have been victims of discrimination and violence, causing them to quit their positions [39,51], which has had a negative impact on medical capacity.

#### Social mobilization

Regarding social mobilization, there was a lack of transparency and empathy in the information provided in the United States [52], which led to public confusion and an underestimation of the virus’s efficacy [42]. Additionally, the slow response of the federal government has weakened the ability of local governments to respond, putting federal and state leadership at odds [52,53]. Some scholars believed that the slow dissemination of relevant information in the United States was mainly to prevent an economic downturn and satisfy public sentiment [42]. Preventive and control measures are difficult to implement in India due to common mass gatherings for religious events and inadequate education about the epidemic. Similarly, in Nigeria, rumours and stigma were widespread, creating barriers to promoting mask-wearing and vaccination. This led to the stigmatization of patients and health workers, as well as causing fear and rejection of isolation among Nigerians [29,43,54].

### International governance

#### Control of international traffic and trade

Regarding the international governance of COVID-19, the United States, Sweden, and Nigeria have faced criticism for their lax management of international travellers. The United States and Sweden were hesitant to enforce strict entry restrictions on Europeans [25,42], while the Nigerian government refused to impose a travel ban on flights originating from severely affected regions, thereby contributing to the expansion of the pandemic [47]. The absence of a travel ban may be due to economic considerations, as disrupting normal communication can harm society as a whole [42].

#### Sharing information and resources

Based on our findings, India and Nigeria have not engaged in significant information and resource sharing. This suggests that developing states may face challenges in providing assistance and support to other nations during global health crises due to their lower levels of economic development. However, the United States, as the most developed state, provided little medical assistance to other states. This situation changed after Joe Biden took office. It implies that a state’s global health governance policies may be closely related to its leader [55]. Maintaining normal trade is crucial during an epidemic as it affects the global supply chain. However, it can be challenging to achieve. India prohibited the export of protective equipment, 26 active pharmaceutical ingredients, and their medicines [10]. The vaccine is the most crucial tool in curbing the pandemic, but its distribution has been inequitable. Low- and middle-income states have not received the necessary vaccines, while developed states have hoarded them instead of disseminating them to those in need [56].

#### Cooperation

Achieving substantive results can be challenging due to the non-binding nature of inter-state cooperation modalities, such as partnerships. Although cooperation is necessary to respond to the pandemic, it is important to acknowledge that human beings are not always rational actors. As stated by one source, ’Instead of acting collectively to save the world, the superpowers fell into a trap of meaningless competition and wasted efforts fighting the virus’ [57]. The United States government politicized and stigmatized public health issues instead of taking full measures to control the epidemic. President Trump repeatedly referred to the coronavirus as the ’Chinese virus’ [58] and portrayed everything related to China as evil. The United States not only criticized China but also announced its withdrawal from the WHO and ended its financial support for the organization. This move has seriously undermined the unity and stability of global health governance [45].

#### Suggestions

When managing infectious diseases, governments should firstly consider unique cultural, social, and economic factors within each state while prioritizing global health due to the borderless nature of viruses.

Before outbreak, it is crucial to strengthen infectious disease surveillance and early warning systems domestically to prevent large-scale outbreaks. This entails establishing robust monitoring networks that integrate data from healthcare facilities, laboratories, and community-based surveillance systems. These systems should employ advanced analytics and machine learning algorithms to detect unusual patterns and provide real-time alerts, enabling rapid response mechanisms. Furthermore, investing in research and development for diagnostic tools and vaccines tailored to local disease profiles can significantly enhance preparedness.

When an infectious disease outbreak occurs, coordinated leadership systems and intermediate strategies maybe effective. Governments should establish multi-sectoral task forces comprising health experts, policymakers, and community leaders to ensure a unified and efficient response. Implementing containment measures such as targeted lockdowns, enhanced hygiene protocols, and social distancing guidelines can mitigate the spread of diseases. Governments should improve risk communication in a timely and transparent manner, based on scientific evidence. This includes regular updates through official channels, clear guidelines for the public, and addressing misinformation actively. To strengthen source control, governments should augment testing capabilities by expanding laboratory networks and utilizing rapid testing technologies. Enhancing hospital capacity, including increasing bed availability, procuring essential medical equipment, and training healthcare workers, is crucial. Implementing necessary closures, such as schools and non-essential businesses, should be done in a phased and strategic manner to balance public health with economic impacts. Additionally, governments should prioritize the protection of vulnerable groups, including the elderly, immunocompromised individuals, and those living in densely populated or underserved areas, by providing them with prioritized access to healthcare services and supplies. Moreover, addressing health inequalities requires a comprehensive approach. Governments should invest in strengthening primary healthcare systems, promoting health education, and improving sanitation and hygiene infrastructures, particularly in rural and marginalized communities.

Internationally, adherence to laws, timely information sharing, and collaboration with organizations such as the WHO are crucial. Developed nations should support developing regions through knowledge transfer, capacity building, and the equitable distribution of vaccines and medical supplies. This includes establishing technology transfer agreements, providing financial aid, and facilitating access to advanced medical research. Efforts should also be made to combat vaccine nationalism and ensure that no country is left behind in the global vaccination drive. To effectively combat infectious diseases and achieve global health goals, it is important for all countries to prioritize cooperation, actively participate in multilateral partnerships, and resolve conflicts diplomatically. This includes refraining from harmful competition, such as hoarding medical resources or engaging in vaccine diplomacy that undermines global solidarity. Instead, fostering a spirit of global citizenship, where nations work collaboratively towards a common health security agenda, is essential for sustainable global health governance.

## Limitations

As the vast majority of the studies included in this review used similar qualitative research methods and presented relevant information in an enumerative manner, all studies included in the review, which are cited in the results and/or discussion sections, are shown through Tables 1 and 2 to facilitate the demonstration of policy differences across countries. This approach differs from the previous characterization of all the studies. The presented research did not show further evidence to suggest that the differences described between the countries were a mere function of the study design and therefore confirmatory. Furthermore, it did not reveal evidence to suggest that the "representative" country selected influenced the findings. It needs more evidence to suggest that if another country from the same category had been selected, the findings would have been similar. Furthermore, the paper is largely descriptive and lacks quantitative analysis to compare the practice of different countries.

## Conclusions

This paper examined the early COVID-19 prevention and control policies implemented by four states’ governments. Notable disparities in the domestic and international governance of COVID-19 were observed among all four states. These disparities may be influenced by different political, economic, cultural, and other factors. To prevent and mitigate the impact of infectious diseases in the future, all states should further prioritize solidarity and cooperation and improve governance domestically and internationally based on their national contexts and global health principles.

## Supporting information

S1 Checklist Preferred Reporting Items for Systematic reviews and Meta-Analyses extension for Scoping Reviews (PRISMA-ScR) checklist. (DOCX)

S1 File Methodological details and S1 and S2 Tables. (DOCX)
