# Supplementary material for: Comparative analysis of state-level policy responses in global health governance: A scoping review using COVID-19 as a case
Source: PLoS One. 2024 Nov 7;19(11):e0313430. doi: 10.1371/journal.pone.0313430 (PMC11542793; doi:10.1371/journal.pone.0313430)
Supplement: S1 File — (DOCX) [file pone.0313430.s002.docx]

Methodological details

A review of peer-reviewed literature was conducted to identify sources that addressed four key concepts: COVID-19, GHG, policy, and representative states (the U.S., Sweden, India and Nigeria). Arksey and O’Malley’s five-stage scoping review framework and PRISMA Extension for Scoping Reviews (PRISMA-ScR) Checklist were employed to guide the review process. The Population, Concept, and Context (PCC) framework for scoping reviews was utilized to organize the findings. The search strategy was developed in consultation with the public health-related librarian at Zhejiang University. Search terms and keywords were first tested in Medline and then applied to other bibliographic databases. Two reviewers independently conducted the literature screening process, reading the title, abstract, and/or full text. In the event of any discrepancies, a third reviewer was consulted to resolve the issue. The results were imported and managed using NoteExpress (Version 3.9.0.9640), and any duplicate references were removed. For official websites, we searched the website of, U.S. Centre for Disease Control and Prevention, Government Offices of Sweden, Ministry of Health and Family Welfare and Nigeria Centre for Disease Control and Prevention.

Search terms and keywords employed in the review

COVID-19: COVID-19 or SARS-CoV-2 or Coronavirus infections or coronavir or corona virus or betacoronavirs or covid19 or covid 19 or ncov or CoV 2 or cov2 or sarscov2 or 2019ncovor sars cov2 or sars-cov-2 or 2019 ncov or novel CoV

GHG: Global Health or government or internationality or international cooperation or global governance or government or collaboration or response or coordination or governanceor government or policy or government or internationality or international cooperationor cooperation or partnership or coordination or leadership or global actors or state actors or non-state actors or global partnerships or global coordination or international obligations or national obligations or international assistance or international cooperation or intergovernmental or multilateral organizations or non-government organizations orinstitutions or governance arrangement

Policy: policy or measure or initiative or strategy or action plan or program or agenda or regulation or legislation or directive or guideline or protocol or blueprint or plan of action or roadmap

Representative states: United States or U.S. or United States of America or USA or America or the States or Sweden or Kingdom of Sweden or India or Republic of India or Bharat or Nigeria or Federal Republic of Nigeria

S1 Table: The details of domestic governance policies and measures in the early stage of the COVID-19 pandemic among four states

| Dimension | Aspect | Details | Key points | the U.S. | Sweden | India | Nigeria |
| --- | --- | --- | --- | --- | --- | --- | --- |
| Infection Prevention and Control | Controlling the source of infection | Detecting cases | Risk screening in key institutions | CDC began health screenings at airports to identify ill travelers returning from Wuhan City | Urged Swedish travelers from China and other hard-hit countries to be aware of symptoms, but not advised to quarantine | NA | Implemented Inter-State border screening in priority areas |
|  |  |  | Risk screening in key populations | Screened travelers from China and referred them to U.S. health departments for oversight of self-monitoring  Travelers with signs or symptoms of illness received a more comprehensive public health assessment.  Conducted testing for individuals in high-risk groups like healthcare workers | Urged travelers to notice symptoms and then tested these with symptoms  Tested sick patients and healthcare staff | Bhilwara continuously followed up on these cases, twice a day, to understand if they were exhibiting symptoms and need further treatment (660) | Implemented mandatory testing for international returnees, suspected cases |
|  |  | Tracing contacts |  | Identified and monitored all persons considered to have had close contact with patients with confirmed COVID-19  States additionally released digital tracing apps because of the deficiency of contact tracers | Implemented contact tracing in the early start and abandoned during community transmission then recommend carrying out trancing contacts by patients themselves | Mobile app for digital contact tracing was launched (636) Conduct close contact tracing and door-to-door testing in the quarantine area (636) | Traced contacts of confirmed cases |
|  |  | Improving detection capability |  | CDC conducted different types of test including RT-PCR, serologic test and viral RNA and antigen test and removed test restrictions.  The Food and Drug Administration (FDA) eased restrictions on the development and use of other test kits and simplified the test approval process to improve the testing speed and capacity Addressed insurance coverage of coronavirus testing and the Paycheck Protection Program which allotted fund to increase testing capacity  Rolled out drive-through testing sites across the country | Most regional laboratories had PCR test in place by the end of March, 2020  Public Health Agency (PHA) presented a national strategy to increase testing  Allocated major resources to enable regions to carry out testing and tracing | Enhanced nucleic acid testing in New Delhi 5893 Expand laboratory testing using Ministry of Health and Family Welfare (MOHFW) and non-ICMR laboratories in many facilities and organizations457 | Designated and strengthened laboratories for COVID-19 testing  Utilized testing laboratories and systems established for other diseases to test SARS-CoV-2  Private laboratories were engaged to enhance the national testing capacity |
|  |  | Locking down risk areas |  | On 16 March, president implemented a 15-day voluntary national lockdown, which extended to 30 April. | No lockdown | A nationwide lockdown was implemented, and the states were later divided into red, orange, and green zones, with the red and orange zones implementing strict "stay-at-home orders" and the green zones allowing related activities (636) | Conducted the lockdown of the three most affected states and closing the Nigerian borders |
|  | Cutting off transmission routes | Managing cases |  | For people who are older or those at high risk of getting very sick from COVID-19, treatment may be available   Recommended people who were sick staying home, preventing COVID-19 from spreading to other people, and following healthcare provider instructions | Self-quarantine | Began to provide free testing and treatment facilities for COVID-19 patients on 30 July 2020 (636) | Isolated confirmed cases. |
|  |  | Managing potential cases |  | Isolated potential patients, and informed public health departments  Recommended staying home when people were ill  Implemented quarantine measures for arriving international travelers with known exposure to locations and settings of concern | Recommended staying at home if symptomatic and avoiding social contacts | Conduct community quarantine on close contacts(636) | Advised people who came back from China in 14 days to isolate themselves and avoid contact with other people  Implemented mandatory supervised isolation for repatriated citizens |
|  |  | Supervising personal protection |  | The federal government issued guidance on hand washing and respiratory etiquette and advised against close contact with those who were sick.   CDC encouraged all high-risk individuals (defined as those over 60 or with underlying chronic health conditions) to avoid face-to-face contact and advised all Americans to wear cloth face coverings in public areas | Recommended avoiding crowding, upholding stringent hand hygiene  Didn't recommend wearing masks outside health care and elderly care  Physical distancing was recommended, only mandatory in bars, restaurants, elderly care homes, and at events | Made it mandatory to wear masks or be arrested and jailed 5893 | Mandatory wearing of masks in public  Encouraged people to maintain social distancing, regular hand washing and use of sanitizers and good reparatory hygiene |
|  |  | Restricting the movement of people |  | CDC informed travelers and clinicians about current health issues outside U.S. and recommended reconsidering the unessential travel The guideline of lockdown advised against discretionary travel  The CDC had a No Sail Order in place | Issued an entry ban for nonessential travel to Sweden from countries outside the EU  Advised against international travel | All forms of transportation services and movement of vehicles were stopped in Bhilwara（660） | Implemented domestic and international travel restriction |
|  |  | Social distancing | Controlling or cancelling mass gatherings | Limited mass gatherings to 10 people during the voluntary lockdown | Issued bans on public gatherings more than 50 together with restrictions for restaurants | Bhilwara imposed a curfew making gatherings of five or more persons unlawful（660） | Limited non-essential activities in priority areas  Prohibited religious and social gatherings involving more than 20 persons |
|  |  |  | Closing recreation places, workplaces, schools | Most states closed all non-essential businesses, restaurants and bars for dine-in seating, and schools | Schools for children up to 16 and public spaces were open   Closed high schools and universities | Industries, shops, malls, and markets across Bhilwara were shut down (660)  Closed all educational institutions (636) | Shut down all schools, government parastatals  Restricted businesses except those involving essential products such as foods, drugs, fuel and gas etc. |
|  |  |  | Issuing a stay-at-home order | 42 states had a mandatory stay-at-home order in place at some point during the national lockdown | Urged people to work from home | Required factories, shops and other entities to adopt a "Work From Home Policy" and pay wages as usual(636) | Issued stay-at-home orders in the FCT, Lagos and Ogun States |
|  | Protecting susceptible populations | Protecting key populations | Protecting the safety of older people, pregnant women, children and other vulnerable groups | Evacuated citizens, residents from foreign hard-hit locations and quarantined them  Collaborations between CDC and health departments focused attention on health equity issues among disproportionately affected populations such as racial and ethnic minorities, frontline workers, the homeless and the person lived in rural and frontier areas | Pinpointed risk groups (people with conditions, people living in nursing home or receiving municipal care) to tailor measures  Banned visits to nursing homes and hospital inpatients  Obligated providers of long-term care to develop safety measures  Urged people with symptoms to avoid working with elderly or people in risk groups  Allocated funds to strengthen care of older people | MoHFW published health advice and guidelines for vulnerable groups （MoHFW) | Government gave directives that reliefs of food materials and money should be distributed to the poor and vulnerable groups |
|  |  |  | Protecting the safety of health workers | Developed infection prevention and control guidance for healthcare providers  Provided training and supplies to health care workers | Staff were informed about how to use protective equipment and have used facemasks throughout the pandemic  Staff were paid more | Insurance Scheme for Health Workers FightingCOVID-19 has been introduced (MoHFW) | NCDC tried to ensure that health workers were provided with the required personal protective equipment  NCDC supported the training of health workers in infection prevention and control |
|  |  | Promoting vaccine |  | Recommended updated COVID-19 vaccine for everyone 6 months and older | Implemented the largest vaccination program in Swedish history  Recommended that everyone aged 50 years and above get vaccinated against COVID-19.   Recommend people aged 18 years and above get vaccinated if they belong to a risk group for severe COVID-19 | Priority was given to key populations such as health workers, and then extended to those over 60 years of age, and to those over 18 years of age in May 2021（679） | Updated the communication strategy to address vaccine uptake and hesitancy. |
|  |  | Sustaining medical services | Setting up COVID-19 designated hospitals | Develop dedicated COVID-19 hospital such as BayPark Hospital (BPH), a 77-bed acute-care hospital |  | Start-up of the Sardar Patel COVID-19 Care Centre, one of the largest hospitals in India and the world to treat COVID-19 patients. | NA |
|  |  |  | Carrying out telemedicine | The Centers for Medicare and Medicaid Services made modifications to facilitate the provision of virtual care for patients who were being advised to not leave their homes. | Family doctors used phone or online consultations to provided patients with services | MoHFW published Telemedicine Practice Guidelines (MoHFW) | NA |
| Overall Strengthening and Optimization | Speed of policy response | Time from the first case to the introduction of a response policy |  | CDC established a 2019-nCoV Incident Management Structure on January 7, 2020 before the first case confirmed on January 20 | The first case of COVID-19 appeared January 31 and COVID-19 was classified as disease dangerous to the public and society | Before theidentification of the first case on January 31, 2020, india issued Travel advisory to travelers visiting China (MoHFW) | The first COVID-19 case was identified on 27 February 2020 and the NCDC activated a Level 3 Emergency Operations Centre (EOC) on the same day |
|  | Collaborative Governance | The form of leadership |  | Established a public health emergency management system with a vertical hierarchy of “federal–state–local”, and a horizontal division of labor between governments, private sectors, and volunteer group   CDC activated its Emergency Operations Center to optimize coordination for domestic and international response efforts | The Public Health Agency of Sweden is the national coordinating body, which has a broad responsibility for public health  Regions of Sweden are autonomous when it comes to management of health care | MoHFW coordinated the awareness efforts at the national level and oversaw the uniform implementation of guidelines at the level of the states, which were held responsible for overseeing the dissemination of information for the containment of disease at regional/district levels The state headquarters provides coordination of technology and resources to local governments, and the local administration acts without any interference   The district administration has coordinated with all line departments | Inaugurated the Presidential Task Force (PTF) on COVID-19 to coordinate the country’s multisectoral intergovernmental response, which included Ministry of Health and NCDC  At state-level, the public health response to COVID-19 is led by State Ministries of Health |
|  |  | Making appropriate modifications to the laws or regulations |  | Telehealth services were built up through waivers granting states relaxation of regulations of virtual visits | Implemented stricter regulations and recommendations due to “corona fatigue”  The Swedish Government decided that the provisions in the Communicable Diseases Act on diseases that pose a danger to the public and to society were to be applied to COVID-19 | The increasing incidents of violence and discrimination against health care professionals led the Indian government to declare any act of such degree a criminal and punishable offense, and through the “Epidemic Diseases (Amendment) Ordinance, 2020 | NA |
|  | Level of emergency response | The level of emergency response |  | Declared a public health emergency when the first case was confirmed on 31 January and a national emergency on 13 March  Various states in the United States have entered a state of emergency, a "disaster state" of the epidemic or a "major disaster state" | On March 10, the Public Health Agency reviewed the alert level and the risk of transmission was upgraded to high for the whole of Sweden. | COVID-19 has been declared a national disaster by the Indian government. | Activated at the highest level of response in the country when the first case was identified |
|  | Basic security | Ensuring the production and supply of daily necessities, medical equipment and production |  | Provided funding for producing and purchasing ventilators and masks  The Defense Production Act was employed a few times  Trump exercised his authority to ramp up the production of much-needed medical supplies | Sweden signed an agreement to enable joint EU-wide procurement of different types of medical counter-measures | Bhilwara ensured door-to-door regular supplies of milk, vegetables, grocery, and other essentials | Personal protective equipment (PPE) and response commodities were deployed across the country |
|  |  | Providing palliative support to individuals and enterprises |  | Paid sick leave, family leave, nutrition assistance, health care, unemployment benefits, and supported coverage for the uninsured,  Provided $2.2 trillion in funding for individuals, small businesses, large corporations, state and local governments, other public health initiatives, and medical intervention like vaccines and rapid testing. | Provided financial support to sick employees and people in multigenerational households  Abolished the qualifying period for benefits  Government paid for sick leave costs rather than companies  Invested 3.8% of GDP to support business | Economic stimulus policies were implemented to improve people's livelihood and stimulate consumption  making masks and sanitizer available and providing free testing and diagnosis. | Conditional cash transferred for vulnerable citizens |
|  |  | Protecting mental health |  | The FDA issued guidance during April 2020 to help expand the availability of digital health therapeutic devices for psychiatric disorders to facilitate consumer and patient use | NA | MoHFW published Guidance for General Medical and pecialised Mental Health Care Settings (MoFHW) | NA |
|  |  | Improving medical care |  | Developed and posted specific guidance for health care settings | Coordinated and transformed the work of healthcare personnel  Allocated funds to raise staff skills | MoHFW published National Guidelines for Infection Prevention and Control in health care facilities (MoHFW) | NCDC developed technical guidelines, response plans and trained health workers across the country on sample collection and testing and clinical management |
|  | Scientific research | Promoting the research of diagnosis and treatment methods, and the development and production of drugs and vaccines |  | In February, the National Institutes of Health (NIH) and FDA were working on development of candidate vaccines and therapeutics for COVID-19  The FDA and CDC greatly simplified the approval process for antiviral drugs and test kits  The Coronavirus Preparedness and Response Supplemental Appropriations Act provided more than US$ 3 billion for research and development of diagnostic tests, therapies, and vaccines | NA | As of May 2021, two vaccines, Covishield and Covaxin, are manufactured in India (https://www.bbc.com/zhongwen/simp/world-57204213） | Nigeria COVID-19 Research Consortium (NCRC) developed and implemented a coordinated research agenda.  Federal Ministry of Health conducted scientific, clinical, anthropological and socio-economic research |
|  | Social mobilization | Engaging in risk communication with the public and controlling rumors |  | The prevention and control knowledge were promoted through websites and public speeches | Held press conferences every weekday Added 17 new languages to the PHA web page on COVID-19 Swedish Civil Contingencies Agency passed on information from agencies and other responsible bodies to the general public and developed national information material | Bhilwara produced daily updates on epidemic progress for the public and reports for authorities | Held daily PTF media briefings   Dynamic listened and managed rumors via media and other social science tools  Engaged communication with affected communities directly or through influencers  Translated COVID-19 information into local languages of vulnerable groups. |
|  |  | Carrying out public health education |  | CDC worked closely with state and local health partners to develop and disseminate information to the public on general prevention | Implemented distance education in upper secondary schools and universities | Public awareness and programs of “do’s and don’ts” for COVID-19 are run at public places. | Issued public health advisory on the website of NCDC  The NCDC worked closely with the Federal Ministry of Information and Culture, as well as the National Orientation Agency to educate Nigerians  Sent out over 100 million text messages to remind Nigerians about measures to protect themselves |
|  |  | Mobilizing the private sector and communities |  | The Defense Production Act, which gives federal agencies the authority to require companies to priorities government contracts for medical supplies  Operation Warp Speed is a program of public-private partnerships that accelerated the development, manufacture, and distribution of COVID-19 diagnostics, therapies, or vaccines. | Interacted with community leaders, representatives of migrant associations, religious leaders and other influencers to reach out with culturally sensitive information | Mobilized appropriate resources from the private sector | Private sectors contributed to several mobile diagnostic laboratories  The organized private sector established the Coalition Against COVID-19 to coordinate their engagement |
|  | Resource adjustment | Expanding the number and capacity of medical institutions |  | Medical-surgical unit were converted to ICU beds  Converted hotels, dormitories, convention centers, and other facilities into makeshift hospitals | Set up field hospitals  Mobilized hospitals to increase beds and coordinated the need for ICU beds at a national level  Converted selected departments to COVID-19 wards | The health infrastructure is ramped up in hospital to expand bed capacity   Private hospitals, train carriages, rooms in hotels, resorts and so on were acquired and converted into quarantine facilities  Built a square cabin hospital in the Bangalore International Exhibition Centre | Designated, strengthened and expanded COVID-19 treatment centers |
|  |  | Supporting hard-hit areas |  | CDC has deployed teams to the U.S. jurisdictions with cases to assist with epidemiologic investigation and to work closely with state and local partners  Distributed Strategic National Stockpile to hard-hit areas | Pinpoint risk areas to tailor measures  The NBHW coordinated material supply to the healthcare among the regions | NA | Deployed Rapid Response Teams (RRTs) |

NA: Not available

The table presents the details of domestic governance policies and measures implemented in the early stage of the COVID-19 pandemic in the four represented states

S2 Table: The details of international governance policies and measures in the early stage of the COVID-19 pandemic among four states

| Aspect | Details | U.S. | Sweden | India | | Nigeria |
| --- | --- | --- | --- | --- | --- | --- |
| Complying with regulations | Providing timely information to WHO | Participated in the development of a new pandemic preparedness instrument and strengthen IHR reporting | Sweden has been in continuous dialogue with WHO’s leadership | NA | | NA |
|  | Reasonable control of international traffic and trade | Issued travel restrictions for non-U.S. citizens or permanent residents arriving from China and later expanded to include other countries with widespread sustained transmission  Issued warnings to avoid nonessential international travel as well as all cruise ship travel  Closed the US borders with Canada and Mexico. | Non-essential travel to other countries is not recommended | Asked the public to avoid unnecessary travel to China Banned Chinese tourists from entering the country  Closed borders and travelers from hard-hit countries will be quarantined for 14 days after entering India Entry of international passengers was prohibited | | Effected restrictions on local and international flights |
|  | Improving the prevention and control capacity of entry points | Deployed CDC staff to CDC quarantine stations at entry ports | NA | Passengers with suspicious symptoms during customs clearance must be forcibly sampled (MoHFW) | | Automated thermal screening at points of entry  Travelers from China were asked questions upon arrival about symptoms of illness and travel history and were advised to contact NCDC if they feel unwell after a trip to Wuhan. |
| Sharing information | Disseminating knowledge, experience, techniques and practices | CDC uploaded the genome of the virus to GenBank and distributed isolates to U.S. public health and academic institutions for additional research, including vaccine development. | Sweden participated in work conducted by the EU Health Security Committee that sought to facilitate the sharing of information and coordination between Member States on questions concerning cross-border health threats | NA | | NA |
|  | Referring to other countries' experience in prevention and control | Participated in the China-WHO Joint expert team and conducted field visits in China  analyzed the experiences of other country to determine the effectiveness of the surveillance and containment measures | NA | NA | | Participated in the China-WHO Joint expert team and conducted field visits in China |
| Sharing resources | Providing assistance and support to affected countries and developing countries | Provided active support to CDC offices in affected countries.  The Coronavirus Preparedness and Response set an amount of US$ 1.25 billion for international assistance, indiplomatic programs, global public health, disaster assistance, or economic support  Project Extension for Community Healthcare Outcomes were used to rapidly disseminate relevant, up-to-date COVID-19–related clinical information to a large, multidisciplinary audience of stakeholders within their healthcare systems. | Donated 722,000 does of COVID-19 vaccine to low- and middle-income countries | Donated masks and other medical supplies to China in Feb 2020 (5893) India committed to delivering nearly 100 million Hydroxychloroquine tablets to Russia | | NA |
|  | Supporting organizations that promote health governance | Supported WHO and other international organizations as they provide guidance for building International Health Regulations (IHR) core capacities. | Sweden contributed both political and financial support to the WHO  Sweden provided core support to many other multilateral organizations including World Bank, the IMF, Gavi and the Vaccine Alliance  Provided millions of SEK to Global Fund, UN’s Central Emergency Response Fund (CERF), UN COVID-19 Response and Recovery Multi-Partner Trust Fund (COVID-19 MPTF) , International Monetary Fund (IMF) Catastrophe Containment and Relief Trust | NA | | NA |
|  | Reducing trade barriers and maintaining trade in essential goods | NA | Sweden worked to ensure the smooth functioning of the European single market and removal of newly imposed export restrictions.   Sweden launched the ‘Trade for Health’ initiative, aimed at removing tariffs and other trade barriers on medical products.   Sweden pushed in the WTO for the elimination of tariffs on environmental goods and services and promoted gender equality in trade | NA | | NA |
|  | Distributing resources such as medicines and vaccines equitably | Biden–Harris administration announced that the USA would provide 80 million vaccine doses to help countries around the world to combat COVID-19 and the USA ensured to work with other partners for equitable access to vaccines, spur production and manufacturing for vaccines and raw materials | Sweden has donated more than 15 million doses and contributed more than SEK 2 billion to global vaccination efforts against COVID-19 | As of February 5, 2021, India has donated and commercially exported vaccines to 17 countries(https://www.bbc.com/zhongwen/trad/world-55948790)  Some smaller donations of vaccines have been granted, and a limited portion has been made available to the Covax Global vaccine sharing program. (https://www.bbc.com/zhongwen/simp/world-57204213) | | NA |
| Strengthening cooperation | Supporting and participating in international research collaborations | Conducted and participated in therapeutic and vaccine clinical trials as appropriate. | NA | India collaborated with US on COVID-19 vaccine development (https://www.bbc.com/zhongwen/simp/world-52447730) | | NA |
|  | Strengthening cooperations with international governmental organizations, international non-governmental organizations and the private sectors | Deployed CDC staff members to work with WHO  Broadened global respiratory surveillance activities by building on and enhancing the Global Influenza Surveillance and Response System (GISRS) in collaboration with WHO | Supported the appeal of UN for a global ceasefire  Sweden worked to fight rumors and disinformation around the COVID-19 virus in cooperation with the EU and other actors | India has partnered with Astrazeneca to produce large quantities of COVID-19 vaccine for the COVAX programme | In February 2020, the Director General of NCDC was part of the WHO mission to China on COVID-19  NCDC Joined WHO/Africa CDC Regional Sequencing Network for COVID-19 and Emerging Pathogens  Collaboration with partners, which facilitated by technical and material support from several local and international partners  NCDC and UNICEF launched chatbot to combat COVID-19 misinformation in Nigeria | |
|  | Promoting partnerships, long-term cooperation mechanisms and cooperation with regional health organizations | CDC leveraged longstanding relationships with U.S. President’s Emergency Plan for AIDS Relief (PEPFAR) clinical partners in multiple countries to support their COVID-19 vaccine administration efforts. | Sweden supported the ‘Team Europe’ approach, which involved extensive collaboration between EU institutions, Member States and financial institutions to support partner countries | On April 28, 2020, heads of BRICS Foreign Ministries held a special meeting on COVID-19 to support the joint establishment of an early warning mechanism for infectious diseases. A fund named the COVID Fund for South Asian Association for Regional Cooperation (SAARC) Countries has been started by SAARC countries to fight COVID-19 | Nigeria led infection prevention and control training to strengthen coronavirus preparedness in Africa in collaboration with the Africa Centre for Disease Control | |

NA: Not available

The table presents the details of domestic governance policies and measures implemented in the early stage of the COVID-19 pandemic in the four represented states
